# Bis[μ-*N*′-isobutyryl-1-oxidonaphtha­lene-2-carbohydrazidato(3-)]dipyridine­tricopper(II)

**DOI:** 10.1107/S1600536809009003

**Published:** 2009-03-25

**Authors:** Xue-Feng Shi, Dacheng Li, Pi-Yong Li, Da-Qi Wang

**Affiliations:** a College of Chemistry and Chemical Engineering, Liaocheng University, Shandong 252059, People’s Republic of China

## Abstract

The complete mol­ecule of the title complex, [Cu_3_(C_15_H_13_N_2_O_3_)_2_(C_5_H_5_N)_2_], is generated by crystallographic twofold symmetry, with the central Cu atom lying on the rotation axis: it is coordinated by two *N*,*O*-bidentate ligands in a *trans*-CuN_2_O_2_ distorted square-planar arrangement. The other Cu atom is coordinated by an *N*,*O*,*O*′-tridentate ligand and a pyridine mol­ecule in a distorted *trans*-CuN_2_O_2_ arrangement. In the crystal structure, a C—H⋯π inter­action occurs.

## Related literature

For related structures, see: Patole *et al.* (2003 ▶); Pouralimardan *et al.* (2007 ▶). For background on C—H⋯π inter­actions, see: Nishio (2004 ▶); Saalfrank & Bernt (1998 ▶).
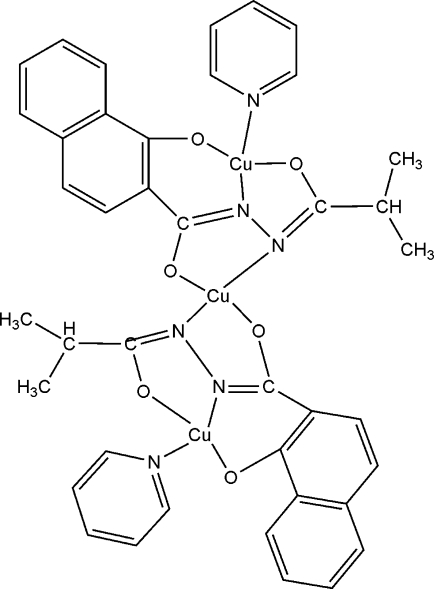


## Experimental

### 

#### Crystal data

[Cu_3_(C_15_H_13_N_2_O_3_)_2_(C_5_H_5_N)_2_]
*M* *_r_* = 887.37
Monoclinic, 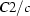

*a* = 23.661 (2) Å
*b* = 13.0521 (18) Å
*c* = 13.3142 (15) Å
β = 113.684 (2)°
*V* = 3765.5 (7) Å^3^
*Z* = 4
Mo *K*α radiation
μ = 1.74 mm^−1^
*T* = 298 K
0.37 × 0.35 × 0.31 mm

#### Data collection

Siemens SMART CCD diffractometer
Absorption correction: multi-scan (*SADABS*; Siemens, 1996 ▶) *T*_min_ = 0.566, *T*_max_ = 0.615 (expected range = 0.537–0.584)
9477 measured reflections
3310 independent reflections
2319 reflections with *I* > 2σ(*I*)
*R*_int_ = 0.050

#### Refinement

*R*[*F* ^2^ > 2σ(*F* ^2^)] = 0.039
*wR*(*F* ^2^) = 0.135
*S* = 1.00
3310 reflections
251 parameters
H-atom parameters constrained
Δρ_max_ = 0.82 e Å^−3^
Δρ_min_ = −0.33 e Å^−3^

### 

Data collection: *SMART* (Siemens, 1996 ▶); cell refinement: *SAINT* (Siemens, 1996 ▶); data reduction: *SAINT*; program(s) used to solve structure: *SHELXS97* (Sheldrick, 2008 ▶); program(s) used to refine structure: *SHELXL97* (Sheldrick, 2008 ▶); molecular graphics: *SHELXTL* (Sheldrick, 2008 ▶); software used to prepare material for publication: *SHELXTL*.

## Supplementary Material

Crystal structure: contains datablocks I, global. DOI: 10.1107/S1600536809009003/hb2917sup1.cif

Structure factors: contains datablocks I. DOI: 10.1107/S1600536809009003/hb2917Isup2.hkl

Additional supplementary materials:  crystallographic information; 3D view; checkCIF report

## Figures and Tables

**Table 1:** Selected bond lengths (Å)

Cu1—O1	1.920 (3)
Cu1—O1^i^	1.920 (3)
Cu1—N2	1.946 (3)
Cu1—N2^i^	1.946 (3)
Cu2—N1	1.884 (3)
Cu2—O2	1.890 (3)
Cu2—O3	1.953 (3)
Cu2—N3	1.975 (3)

Symmetry code: (i) 
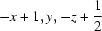
.

**Table 2:** Hydrogen-bond geometry (Å, °) *Cg*1 is the centroid of the C2–C7 ring.

*D*—H⋯*A*	*D*—H	H⋯*A*	*D*⋯*A*	*D*—H⋯*A*
C17—H17⋯*Cg*1^ii^	0.93	2.53	3.362 (4)	150

Symmetry code: (ii) 
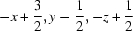
.

**Fig. 1.**
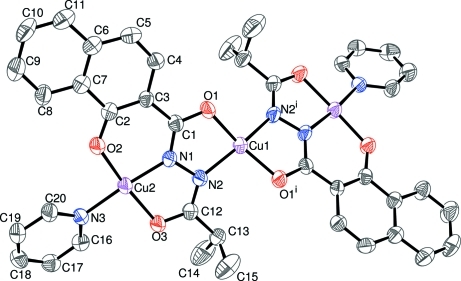
The molecular structure of (I) showing 40% probability displacement ellipsoids. H atoms have been omitted for clarity. Symmetry code: (i) 1–x, y, 1/2–z.

**Fig. 2.**
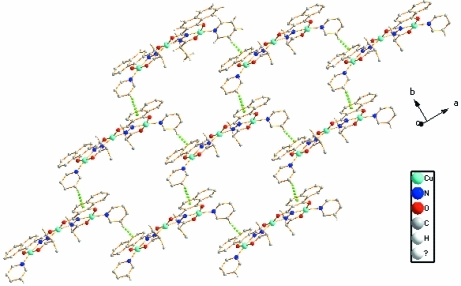
View of the two-dimensional network structure in (I). Intermolecular C—H···π are shown as dashed lines. Most of H atoms are omitted.

## supplementary crystallographic information

### Comment

A large number of aroylhydrazine complexes have been prepared and studied due to their diverse molecular architectures and quite interesting chemical properties (Patole *et al.*,  2003; Pouralimardan *et al.*, 2007). However, researches on the copper(II) complexes with N-isobutyryl-1-hydroxy-2-naphthalenecarbohydrazide have not reported. So we have synthesized a new complex, (I), (Fig. 1), which has been characterized by X-ray diffraction and elemental analysis.  The molecule of (I) contains three copper(II), two ligand molecules, and two pyridine molecules.  Both copper centres adopt distorted square planar trans-CuN_2_O_2_ arrangements. The triple-deprotonated *N*-isobutyryl-1-hydroxy-2-naphthalenecarbohydrazide bridges the metal ions using a hydrazide N—N group and formed the trinuclear copper complex. In the crystal packing, the complex molecules are linked into two-dimensional network by intermolecular C—H···π interactions (Fig. 2) (Saalfrank & Bernt, 1998; Nishio, 2004).

### Experimental

Isobutyric anhydride (0.632 g, 4 mmol) and 1-hydroxy-2-naphthalenecarbohydrazide (0.808 g, 4 mmol) were added to 40 ml of chloroform at ice-water bath. The reaction mixture was slowly warmed to room temperature and stirred for 24 h. After overnight refrigeration, the resulting white precipitate was filtered and rinsed with chloroform and diethyl ether (1.02 g, 93.57% yield).  A solution of CuNO_3_(0.04 g,0.2 mmol) in methanol (10 ml) was added to a mixture of N-isobutyryl-1-hydroxy-2-naphthalenecarbohydrazide (0.055 g, 0.2 mmol) and sodium methylate (0.0324 g, 0.6 mmol) in pyridine (10 ml). A green solution was obtained after refluxing for 3 h. After being filtrated, dimethyl ether was slowly diffused into the filtrate, and green blocks of (I) were obtained after two weeks. Elemental analysis calculated for C_40_H_36_N_6_O_6_Cu_3_: C, 54.09; H, 4.05; O, 10.78; N, 9.43. Found (%): C, 54.12; H, 4.06; O, 10.82; N, 9.47

### Refinement

The C-bound H atoms were positioned with idealized geometry (C—H = 0.93–0.98Å) and refined as riding with *U*_iso_(H) = 1.2 *U*_eq_(C) or 1.5U_eq_(methyl C).

### Crystal data

**Table:**

[Cu_3_(C_15_H_13_N_2_O_3_)_2_(C_5_H_5_N)_2_]	*F*(000) = 1812
*M**_r_* = 887.37	*D*_x_ = 1.565 Mg m^−^^3^*D*_m_ = 1.565 Mg m^−^^3^*D*_m_ measured by not measured
Monoclinic, *C*2/*c*	Mo *K*α radiation, λ = 0.71073 Å
Hall symbol: -C 2yc	Cell parameters from 3140 reflections
*a* = 23.661 (2) Å	θ = 2.7–26.3°
*b* = 13.0521 (18) Å	µ = 1.74 mm^−^^1^
*c* = 13.3142 (15) Å	*T* = 298 K
β = 113.684 (2)°	Block, green
*V* = 3765.5 (7) Å^3^	0.37 × 0.35 × 0.31 mm
*Z* = 4	

### Data collection

**Table:**

Siemens SMART CCD diffractometer	3310 independent reflections
Radiation source: fine-focus sealed tube	2319 reflections with *I* > 2σ(*I*)
graphite	*R*_int_ = 0.050
ω scans	θ_max_ = 25.0°, θ_min_ = 1.8°
Absorption correction: multi-scan (*SADABS*; Siemens, 1996)	*h* = −28→27
*T*_min_ = 0.566, *T*_max_ = 0.615	*k* = −14→15
9477 measured reflections	*l* = −13→15

### Refinement

**Table:**

Refinement on *F*^2^	Primary atom site location: structure-invariant direct methods
Least-squares matrix: full	Secondary atom site location: difference Fourier map
*R*[*F*^2^ > 2σ(*F*^2^)] = 0.039	Hydrogen site location: inferred from neighbouring sites
*wR*(*F*^2^) = 0.135	H-atom parameters constrained
*S* = 1.00	*w* = 1/[σ^2^(*F*_o_^2^) + (0.079*P*)^2^ + 4.0071*P*] where *P* = (*F*_o_^2^ + 2*F*_c_^2^)/3
3310 reflections	(Δ/σ)_max_ = 0.001
251 parameters	Δρ_max_ = 0.82 e Å^−^^3^
0 restraints	Δρ_min_ = −0.33 e Å^−^^3^

### Fractional atomic coordinates and isotropic or equivalent isotropic displacement parameters (Å^2^)

**Table:**

	*x*	*y*	*z*	*U*_iso_*/*U*_eq_	
Cu1	0.5000	0.86302 (6)	0.2500	0.0410 (2)	
Cu2	0.68569 (2)	0.80745 (4)	0.23216 (4)	0.0404 (2)	
N1	0.62147 (15)	0.8496 (3)	0.2723 (3)	0.0401 (8)	
N2	0.56137 (15)	0.8380 (3)	0.1900 (3)	0.0425 (9)	
N3	0.74587 (15)	0.7504 (3)	0.1787 (3)	0.0386 (8)	
O1	0.57183 (12)	0.8896 (2)	0.3816 (2)	0.0446 (7)	
O2	0.74777 (12)	0.8431 (2)	0.3698 (2)	0.0442 (7)	
O3	0.61596 (13)	0.7851 (3)	0.0920 (2)	0.0523 (8)	
C1	0.62281 (18)	0.8758 (3)	0.3692 (3)	0.0363 (9)	
C2	0.73934 (18)	0.8742 (3)	0.4567 (3)	0.0361 (9)	
C3	0.68203 (18)	0.8912 (3)	0.4623 (3)	0.0341 (9)	
C4	0.68036 (19)	0.9281 (3)	0.5618 (3)	0.0399 (10)	
H4	0.6421	0.9386	0.5645	0.048*	
C5	0.7314 (2)	0.9484 (3)	0.6518 (3)	0.0440 (11)	
H5	0.7278	0.9733	0.7144	0.053*	
C6	0.7909 (2)	0.9322 (3)	0.6522 (3)	0.0421 (10)	
C7	0.79500 (19)	0.8936 (3)	0.5546 (3)	0.0398 (10)	
C8	0.85377 (19)	0.8776 (4)	0.5545 (4)	0.0503 (11)	
H8	0.8570	0.8522	0.4919	0.060*	
C9	0.9059 (2)	0.8990 (5)	0.6448 (4)	0.0674 (15)	
H9	0.9443	0.8885	0.6426	0.081*	
C10	0.9026 (2)	0.9367 (4)	0.7415 (4)	0.0655 (15)	
H10	0.9385	0.9507	0.8029	0.079*	
C11	0.8464 (2)	0.9522 (4)	0.7439 (4)	0.0559 (13)	
H11	0.8443	0.9767	0.8080	0.067*	
C12	0.56432 (19)	0.8035 (4)	0.0990 (4)	0.0451 (11)	
C13	0.5056 (2)	0.7872 (4)	−0.0027 (4)	0.0548 (13)	
H13	0.4703	0.7944	0.0176	0.066*	
C14	0.5046 (3)	0.6793 (5)	−0.0479 (5)	0.090 (2)	
H14A	0.5318	0.6765	−0.0854	0.135*	
H14B	0.4634	0.6627	−0.0982	0.135*	
H14C	0.5180	0.6310	0.0114	0.135*	
C15	0.5004 (3)	0.8681 (5)	−0.0879 (5)	0.0862 (19)	
H15A	0.5084	0.9344	−0.0539	0.129*	
H15B	0.4595	0.8668	−0.1449	0.129*	
H15C	0.5299	0.8541	−0.1188	0.129*	
C16	0.7284 (2)	0.6777 (3)	0.0995 (3)	0.0414 (10)	
H16	0.6872	0.6577	0.0689	0.050*	
C17	0.7684 (2)	0.6324 (3)	0.0624 (4)	0.0468 (11)	
H17	0.7548	0.5822	0.0084	0.056*	
C18	0.8288 (2)	0.6624 (4)	0.1064 (4)	0.0585 (13)	
H18	0.8571	0.6324	0.0829	0.070*	
C19	0.8476 (2)	0.7379 (5)	0.1861 (4)	0.0604 (13)	
H19	0.8883	0.7605	0.2160	0.072*	
C20	0.8046 (2)	0.7787 (4)	0.2200 (4)	0.0489 (11)	
H20	0.8174	0.8285	0.2745	0.059*	

### Atomic displacement parameters (Å^2^)

**Table:**

	*U*^11^	*U*^22^	*U*^33^	*U*^12^	*U*^13^	*U*^23^
Cu1	0.0270 (4)	0.0562 (5)	0.0400 (4)	0.000	0.0138 (3)	0.000
Cu2	0.0282 (3)	0.0537 (4)	0.0391 (3)	−0.0004 (2)	0.0133 (2)	−0.0070 (2)
N1	0.0243 (17)	0.058 (2)	0.0350 (19)	−0.0019 (16)	0.0083 (15)	−0.0053 (17)
N2	0.0269 (18)	0.062 (2)	0.0371 (19)	−0.0036 (17)	0.0110 (16)	−0.0097 (18)
N3	0.0349 (19)	0.044 (2)	0.0392 (19)	0.0012 (16)	0.0175 (16)	−0.0024 (17)
O1	0.0286 (15)	0.0644 (19)	0.0435 (17)	−0.0017 (14)	0.0172 (13)	−0.0100 (15)
O2	0.0308 (15)	0.0602 (19)	0.0417 (17)	−0.0003 (14)	0.0146 (13)	−0.0083 (15)
O3	0.0291 (16)	0.088 (2)	0.0386 (17)	0.0002 (16)	0.0123 (13)	−0.0135 (16)
C1	0.031 (2)	0.039 (2)	0.041 (2)	−0.0013 (18)	0.0161 (18)	−0.0011 (19)
C2	0.034 (2)	0.032 (2)	0.040 (2)	−0.0022 (17)	0.0121 (18)	0.0006 (18)
C3	0.029 (2)	0.034 (2)	0.037 (2)	−0.0010 (17)	0.0120 (17)	−0.0024 (18)
C4	0.039 (2)	0.042 (2)	0.040 (2)	−0.0004 (19)	0.016 (2)	−0.0016 (19)
C5	0.057 (3)	0.041 (2)	0.034 (2)	−0.003 (2)	0.017 (2)	−0.0002 (19)
C6	0.046 (3)	0.032 (2)	0.041 (2)	−0.0017 (19)	0.011 (2)	0.0039 (19)
C7	0.040 (2)	0.032 (2)	0.043 (2)	−0.0017 (18)	0.013 (2)	0.0008 (19)
C8	0.034 (2)	0.059 (3)	0.053 (3)	0.002 (2)	0.012 (2)	−0.008 (2)
C9	0.037 (3)	0.089 (4)	0.066 (3)	0.001 (3)	0.009 (3)	−0.006 (3)
C10	0.041 (3)	0.072 (4)	0.058 (3)	−0.002 (3)	−0.007 (2)	−0.004 (3)
C11	0.057 (3)	0.054 (3)	0.043 (3)	−0.003 (2)	0.006 (2)	−0.002 (2)
C12	0.031 (2)	0.064 (3)	0.040 (2)	−0.001 (2)	0.0130 (19)	−0.001 (2)
C13	0.031 (2)	0.088 (4)	0.038 (2)	0.001 (2)	0.008 (2)	−0.009 (3)
C14	0.078 (4)	0.082 (4)	0.070 (4)	−0.011 (3)	−0.012 (3)	−0.016 (3)
C15	0.074 (4)	0.084 (4)	0.064 (4)	−0.006 (3)	−0.009 (3)	0.004 (3)
C16	0.042 (2)	0.038 (2)	0.044 (2)	−0.0023 (19)	0.018 (2)	0.001 (2)
C17	0.056 (3)	0.040 (3)	0.046 (3)	0.004 (2)	0.022 (2)	−0.002 (2)
C18	0.057 (3)	0.070 (3)	0.055 (3)	0.024 (3)	0.031 (3)	0.002 (3)
C19	0.035 (3)	0.089 (4)	0.056 (3)	0.005 (3)	0.017 (2)	−0.003 (3)
C20	0.035 (2)	0.062 (3)	0.047 (3)	0.000 (2)	0.014 (2)	−0.008 (2)

### Geometric parameters (Å, °)

**Table:**

Cu1—O1	1.920 (3)	C8—C9	1.361 (6)
Cu1—O1^i^	1.920 (3)	C8—H8	0.9300
Cu1—N2	1.946 (3)	C9—C10	1.409 (7)
Cu1—N2^i^	1.946 (3)	C9—H9	0.9300
Cu2—N1	1.884 (3)	C10—C11	1.360 (7)
Cu2—O2	1.890 (3)	C10—H10	0.9300
Cu2—O3	1.953 (3)	C11—H11	0.9300
Cu2—N3	1.975 (3)	C12—C13	1.516 (6)
N1—C1	1.323 (5)	C13—C15	1.518 (8)
N1—N2	1.412 (5)	C13—C14	1.529 (8)
N2—C12	1.320 (5)	C13—H13	0.9800
N3—C20	1.326 (5)	C14—H14A	0.9600
N3—C16	1.354 (5)	C14—H14B	0.9600
O1—C1	1.294 (5)	C14—H14C	0.9600
O2—C2	1.315 (5)	C15—H15A	0.9600
O3—C12	1.285 (5)	C15—H15B	0.9600
C1—C3	1.465 (5)	C15—H15C	0.9600
C2—C3	1.405 (5)	C16—C17	1.365 (6)
C2—C7	1.455 (6)	C16—H16	0.9300
C3—C4	1.425 (6)	C17—C18	1.367 (7)
C4—C5	1.342 (6)	C17—H17	0.9300
C4—H4	0.9300	C18—C19	1.384 (7)
C5—C6	1.423 (6)	C18—H18	0.9300
C5—H5	0.9300	C19—C20	1.374 (6)
C6—C11	1.412 (6)	C19—H19	0.9300
C6—C7	1.433 (6)	C20—H20	0.9300
C7—C8	1.406 (6)		
			
O1—Cu1—O1^i^	159.21 (19)	C7—C8—H8	119.5
O1—Cu1—N2	82.65 (13)	C8—C9—C10	121.0 (5)
O1^i^—Cu1—N2	100.87 (13)	C8—C9—H9	119.5
O1—Cu1—N2^i^	100.87 (13)	C10—C9—H9	119.5
O1^i^—Cu1—N2^i^	82.65 (13)	C11—C10—C9	119.2 (4)
N2—Cu1—N2^i^	160.7 (2)	C11—C10—H10	120.4
N1—Cu2—O2	93.11 (13)	C9—C10—H10	120.4
N1—Cu2—O3	81.17 (13)	C10—C11—C6	122.0 (5)
O2—Cu2—O3	173.00 (13)	C10—C11—H11	119.0
N1—Cu2—N3	172.94 (14)	C6—C11—H11	119.0
O2—Cu2—N3	92.88 (13)	O3—C12—N2	122.2 (4)
O3—Cu2—N3	93.10 (13)	O3—C12—C13	117.8 (4)
C1—N1—N2	114.0 (3)	N2—C12—C13	120.0 (4)
C1—N1—Cu2	130.2 (3)	C12—C13—C15	109.9 (4)
N2—N1—Cu2	115.1 (3)	C12—C13—C14	110.1 (4)
C12—N2—N1	109.9 (3)	C15—C13—C14	111.2 (5)
C12—N2—Cu1	139.2 (3)	C12—C13—H13	108.5
N1—N2—Cu1	110.4 (2)	C15—C13—H13	108.5
C20—N3—C16	117.3 (4)	C14—C13—H13	108.5
C20—N3—Cu2	122.2 (3)	C13—C14—H14A	109.5
C16—N3—Cu2	120.4 (3)	C13—C14—H14B	109.5
C1—O1—Cu1	112.8 (3)	H14A—C14—H14B	109.5
C2—O2—Cu2	126.6 (3)	C13—C14—H14C	109.5
C12—O3—Cu2	111.4 (3)	H14A—C14—H14C	109.5
O1—C1—N1	120.1 (4)	H14B—C14—H14C	109.5
O1—C1—C3	119.8 (4)	C13—C15—H15A	109.5
N1—C1—C3	120.1 (3)	C13—C15—H15B	109.5
O2—C2—C3	125.9 (4)	H15A—C15—H15B	109.5
O2—C2—C7	116.0 (4)	C13—C15—H15C	109.5
C3—C2—C7	118.1 (4)	H15A—C15—H15C	109.5
C2—C3—C4	119.4 (4)	H15B—C15—H15C	109.5
C2—C3—C1	123.3 (4)	N3—C16—C17	123.1 (4)
C4—C3—C1	117.3 (3)	N3—C16—H16	118.5
C5—C4—C3	123.1 (4)	C17—C16—H16	118.5
C5—C4—H4	118.5	C16—C17—C18	118.6 (4)
C3—C4—H4	118.5	C16—C17—H17	120.7
C4—C5—C6	120.6 (4)	C18—C17—H17	120.7
C4—C5—H5	119.7	C17—C18—C19	119.4 (4)
C6—C5—H5	119.7	C17—C18—H18	120.3
C11—C6—C5	123.4 (4)	C19—C18—H18	120.3
C11—C6—C7	118.2 (4)	C20—C19—C18	118.4 (5)
C5—C6—C7	118.4 (4)	C20—C19—H19	120.8
C8—C7—C6	118.6 (4)	C18—C19—H19	120.8
C8—C7—C2	120.9 (4)	N3—C20—C19	123.2 (4)
C6—C7—C2	120.5 (4)	N3—C20—H20	118.4
C9—C8—C7	121.0 (5)	C19—C20—H20	118.4
C9—C8—H8	119.5		
			
O2—Cu2—N1—C1	−10.5 (4)	N1—C1—C3—C4	174.6 (4)
O3—Cu2—N1—C1	173.6 (4)	C2—C3—C4—C5	0.4 (6)
O2—Cu2—N1—N2	179.6 (3)	C1—C3—C4—C5	−177.8 (4)
O3—Cu2—N1—N2	3.6 (3)	C3—C4—C5—C6	−0.9 (7)
C1—N1—N2—C12	−174.6 (4)	C4—C5—C6—C11	179.9 (4)
Cu2—N1—N2—C12	−3.0 (5)	C4—C5—C6—C7	−0.1 (6)
C1—N1—N2—Cu1	−0.9 (5)	C11—C6—C7—C8	−0.1 (6)
Cu2—N1—N2—Cu1	170.69 (17)	C5—C6—C7—C8	179.8 (4)
O1—Cu1—N2—C12	172.5 (5)	C11—C6—C7—C2	−178.4 (4)
O1^i^—Cu1—N2—C12	−28.3 (5)	C5—C6—C7—C2	1.6 (6)
N2^i^—Cu1—N2—C12	70.6 (5)	O2—C2—C7—C8	−1.0 (6)
O1—Cu1—N2—N1	1.6 (3)	C3—C2—C7—C8	179.8 (4)
O1^i^—Cu1—N2—N1	160.8 (3)	O2—C2—C7—C6	177.1 (4)
N2^i^—Cu1—N2—N1	−100.4 (3)	C3—C2—C7—C6	−2.1 (6)
O2—Cu2—N3—C20	−23.5 (4)	C6—C7—C8—C9	−0.4 (7)
O3—Cu2—N3—C20	152.8 (4)	C2—C7—C8—C9	177.8 (5)
O2—Cu2—N3—C16	154.6 (3)	C7—C8—C9—C10	0.7 (8)
O3—Cu2—N3—C16	−29.1 (3)	C8—C9—C10—C11	−0.3 (9)
O1^i^—Cu1—O1—C1	−103.3 (3)	C9—C10—C11—C6	−0.3 (8)
N2—Cu1—O1—C1	−2.0 (3)	C5—C6—C11—C10	−179.4 (5)
N2^i^—Cu1—O1—C1	158.7 (3)	C7—C6—C11—C10	0.5 (7)
N1—Cu2—O2—C2	6.4 (3)	Cu2—O3—C12—N2	3.1 (6)
N3—Cu2—O2—C2	−169.9 (3)	Cu2—O3—C12—C13	−178.6 (3)
N1—Cu2—O3—C12	−3.6 (3)	N1—N2—C12—O3	−0.2 (6)
N3—Cu2—O3—C12	172.2 (3)	Cu1—N2—C12—O3	−171.2 (3)
Cu1—O1—C1—N1	2.2 (5)	N1—N2—C12—C13	−178.4 (4)
Cu1—O1—C1—C3	−179.0 (3)	Cu1—N2—C12—C13	10.7 (8)
N2—N1—C1—O1	−0.8 (6)	O3—C12—C13—C15	−69.6 (6)
Cu2—N1—C1—O1	−170.8 (3)	N2—C12—C13—C15	108.6 (5)
N2—N1—C1—C3	−179.6 (3)	O3—C12—C13—C14	53.2 (6)
Cu2—N1—C1—C3	10.4 (6)	N2—C12—C13—C14	−128.5 (5)
Cu2—O2—C2—C3	−3.0 (6)	C20—N3—C16—C17	0.9 (6)
Cu2—O2—C2—C7	177.9 (3)	Cu2—N3—C16—C17	−177.3 (3)
O2—C2—C3—C4	−178.1 (4)	N3—C16—C17—C18	−0.7 (7)
C7—C2—C3—C4	1.1 (6)	C16—C17—C18—C19	−0.5 (7)
O2—C2—C3—C1	0.1 (7)	C17—C18—C19—C20	1.4 (8)
C7—C2—C3—C1	179.2 (4)	C16—N3—C20—C19	0.0 (7)
O1—C1—C3—C2	177.6 (4)	Cu2—N3—C20—C19	178.2 (4)
N1—C1—C3—C2	−3.6 (6)	C18—C19—C20—N3	−1.2 (8)
O1—C1—C3—C4	−4.3 (6)		

Symmetry codes: (i) −*x*+1, *y*, −*z*+1/2.

### Hydrogen-bond geometry (Å, °)

**Table:**

*D*—H···*A*	*D*—H	H···*A*	*D*···*A*	*D*—H···*A*
C17—H17···Cg1^ii^	0.93	2.53	3.362 (4)	150

Symmetry codes: (ii) −*x*+3/2, *y*−1/2, −*z*+1/2.
